# Comparação entre os Efeitos da Ingestão de Sal do Himalaia e de Sal Comum sobre os Valores de Sódio Urinário e Pressão Arterial em Indivíduos Hipertensos

**DOI:** 10.36660/abc.20210069

**Published:** 2022-01-11

**Authors:** Isabela P. Loyola, Mauri Félix de Sousa, Thiago Veiga Jardim, Marcela M. Mendes, Weimar Kunz Sebba Barroso, Ana Luiza Lima Sousa, Paulo César B. Veiga Jardim

**Affiliations:** 1 Liga de Hipertensão Arterial Universidade Federal de Goiás Goiânia GO Brasil Liga de Hipertensão Arterial - Universidade Federal de Goiás, Goiânia, GO – Brasil; 2 Hospital das Clínicas Universidade Federal de Goiás Goiânia GO Brasil Hospital das Clínicas - Universidade Federal de Goiás, Goiânia, GO – Brasil; 3 Departamento de Nutrição Faculdade de Ciências da Saúde de Brasília Brasília DF Brasil Departamento de Nutrição - Faculdade de Ciências da Saúde de Brasília, Brasília, DF – Brasil

**Keywords:** Pressão Arterial, Hipertensão, Doenças Cardiovasculares, Fatores de Risco, Cloreto de Sódio, Sódio na Dieta, Urinalise

## Abstract

**Fundamento:**

O sal do Himalaia (SH) tornou-se uma alternativa popular para o sal de mesa (SM) devido às suas alegações de benefícios à saúde, principalmente para indivíduos com hipertensão arterial. Porém, apesar do aumento do consumo de SH, ainda faltam evidências clínicas que sustentem a recomendação de seu consumo por profissionais de saúde.

**Objetivo:**

Este estudo teve como objetivo comparar o impacto da ingestão de SH e SM sobre a pressão arterial sistólica (PAS), pressão arterial diastólica (PAD) e concentração de sódio urinário em indivíduos com PA.

**Métodos:**

Este estudo recrutou 17 pacientes do sexo feminino com hipertensão arterial que comiam fora de casa no máximo uma vez por semana. Os participantes foram divididos aleatoriamente em dois grupos, para receber e consumir SH ou SM. Antes e depois de cada intervenção, os participantes tiveram sua pressão arterial medida e urina coletada para análise mineral. Um valor de p <0,05 foi considerado estatisticamente significativo.

**Resultados:**

Não houve diferenças estatisticamente significativas antes e depois da intervenção SH para PAD (70 mmHg vs. 68,5 mmHg; p = 0,977), PAS (118,5 mmHg vs. 117,5 mmHg; p = 0,932) e concentração urinária de sódio (151 mEq / 24h vs. 159 mEq / 24; p = 0,875). Além disso, a análise entre os grupos não mostrou diferenças significativas após a intervenção em relação a PAS (117 mmHg vs 119 mmHg; p = 0,908), PAD (68,5 mmHg vs 71 mmHg; p = 0,645) ou concentração urinária de sódio (159 mEq / 24h vs 155 mEq / 24h; p = 0,734).

**Conclusão:**

Este estudo sugere que não há diferenças significativas no impacto do consumo de SH em relação ao SM na PA e concentração urinária de sódio em indivíduos com hipertensão arterial.

## Introdução

A hipertensão (HTN) é um dos principais fatores de risco para doença cardiovascular (DCV), e afeta mais de 35% da população brasileira acima de 40 anos de idade.^1^ Já está bem estabelecido que o tratamento da HTN pode reduzir o risco de eventos cardiovasculares e, por isso, essa medida é considerada uma das principais estratégias na saúde pública para o controle das DCVs.

A ingestão de sódio é um fator de risco modificável chave na HTN.^2^ Estudos mostram que uma elevada ingestão de sódio está associada a níveis mais elevados de pressão sanguínea, e uma ingestão baixa ou moderada pode estar associada a níveis mais baixos.^2-4^ A Organização Mundial de Saúde (OMS) recomenda atualmente uma ingestão de sódio de 2g por dia;^5^ contudo, em muitos países, o consumo de sal é mais que o dobro.^6^ No Brasil, por exemplo, a média de consumo de sódio é de 4,7 gramas por dia, sendo a maioria originária do sal de mesa (SM) e temperos (74,4%).^7^

Nesse contexto, o sal do Himalaia (SH) tornou-se uma alternativa popular para o SM tradicional, particularmente para indivíduos hipertensos. As mídias sociais tornaram-se parte do cenário da saúde pública, sendo usadas para acessar, compartilhar, e espalhar informações médicas, e responsáveis por mudanças recentes no comportamento em saúde. Nesse contexto de consumo excessivo de mídia, alavancado pelo aumento de propagandas de alimentos via mídias sociais, muitos benefícios à saúde foram atribuídos ao SH, sem evidência científica robusta, contribuindo para a moda do SH.^8^

Aqueles que defendem o consumo do SH para o controle da HTN baseiam-se no fato de o sal não ser refinado e seus efeitos benéficos. A explicação é que, diferentemente do sal tradicional, o SH reteria uma concentração maior de minerais tais como ferro, magnésio, cálcio, zinco, e potássio, os quais são inversamente associados com valores de pressão arterial.^9-11^

Apesar do aumento no consumo do SH e seus supostos benefícios à saúde, ainda há pouca evidência científica que apoiem recomendações clínicas por profissionais da saúde. Assim, o presente estudo teve como objetivo comparar o impacto do SH e do SM sobre a pressão arterial, e sobre concentrações urinárias de sódio e potássio de indivíduos com hipertensão arterial.

## Métodos

### Delineamento do estudo

Este estudo foi um ensaio randomizado do tipo crossover que comparou os efeitos da ingestão de SH e de SM sobre as concentrações de sódio urinário e da pressão sanguínea de indivíduos hipertensos. Foram recrutadas mulheres hipertensas com idade entre 40 e 65 anos de idade a partir de um serviço multidisciplinar para o manejo da HTN. Os critérios de inclusão foram: residir em uma região metropolitana de uma cidade brasileira, sem mudanças no tratamento medicamentoso para HTN por no mínimo 60 dias.

Os cálculos foram baseados em dados prévios sobre os efeitos da redução da ingestão de sódio sobre a pressão arterial.^3^ O tamanho amostral foi calculado para comparação das médias, considerando um tamanho do efeito de 1,56,^3^ alfa de 0,05 e poder do teste (1-β) de 90%, e o resultado foi de 10 participantes em cada grupo.

Foram excluídos pacientes com insuficiência cardíaca, acidente vascular cerebral nos últimos seis meses, infarto agudo do miocárdio nos últimos três meses, diabetes mellitus não controlada (hemoglobina glicada acima de 8%), doença hepática, hipotiroidismo, doença renal crônica, doenças psiquiátricas não estabilizadas, usuários de drogas ilícitas, e alcóolatras, bem como pacientes que, mais que uma vez por semana, preparavam suas refeições utilizando um sal diferente daquele fornecido no estudo.

O estudo foi aprovado pelo Comitê de Ética do Hospital das Clínicas de uma universidade brasileira (069428/2017), e todos os pacientes assinaram o termo de consentimento. O estudo foi conduzido seguindo a Resolução número 446 de 2012.^12^

**Figura 1:**
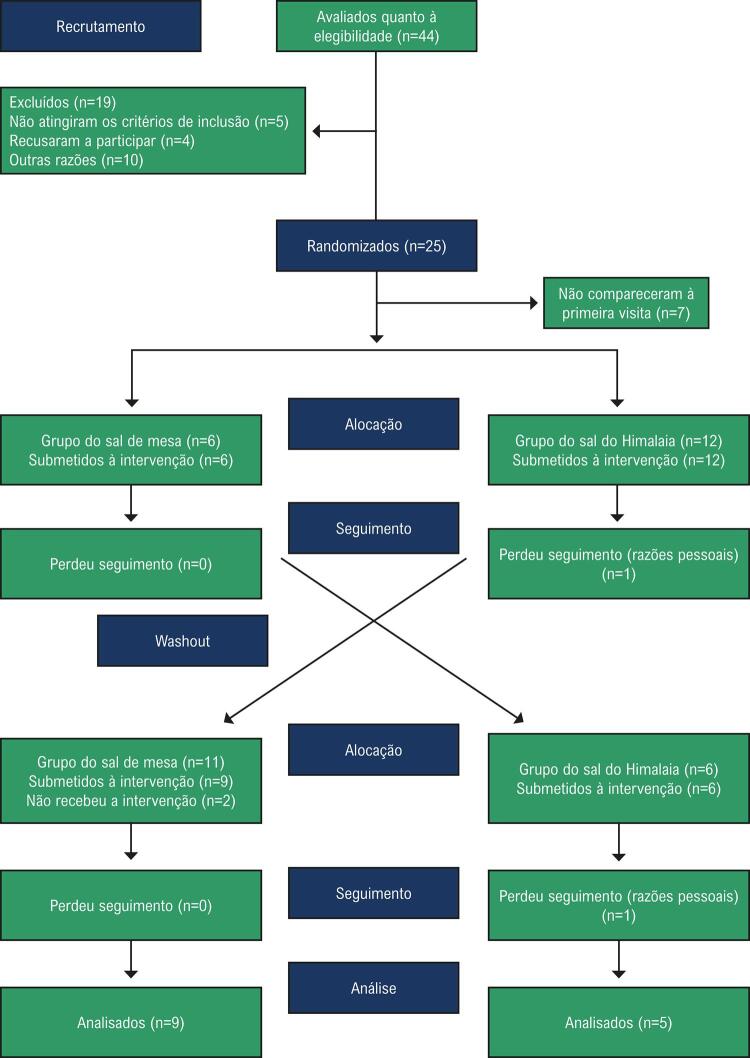
– *Fluxograma da randomização dos pacientes.*

Antes e após cada intervenção (SH e SM), os participantes compareceram a duas visitas, com um intervalo de 3-4 dias entre elas, conduzidas pelo mesmo pesquisador. Antes do início da intervenção, exames bioquímicos foram solicitados aos participantes que não possuíam registros de exames recentes, e medidas antropométricas (peso, altura, e circunferência da cintura), e características demográficas foram coletadas de todos os participantes incluídos no estudo. Na primeira visita, os participantes foram aleatoriamente alocados a utilizar SH ou SM (Figura 1). Após quatro semanas de intervenção, e mais duas semanas livres (*washout*), os participantes foram passaram a receber o sal alternativo por mais quatro semanas de intervenção. Durante o período de *washout*, os participantes foram orientados a manter sua dieta usual e consumirem o sal a que estavam habituados.

Ainda, antes e após cada intervenção, foi fornecido a cada participante um aparelho de pressão arterial para a realização das medidas, bem como um frasco coletor de urina para a coleta de urina em 24 horas. Após três a quatro dias, os participantes retornaram ao centro de pesquisa com o aparelho de pressão e a urina coletada.

### Composição do sal

Nós analisamos nove amostras de SH e três amostras do SM obtidas de mercados de uma região metropolitana no Brasil, para verificar a iodação e concentração de minerais. Todas as amostras analisadas foram fortificadas com iodo.

A marca de SH cujo teor de sódio foi o mais próximo da média obtida de todas as amostras de SH foi escolhida para a intervenção (SH da Intervenção: 371,92 mg de sódio/g, 1,8 mg de potássio/g, 1,7 de magnésio/g, e 25,1 mcg de iodo/g), e a marca de SM escolhida foi a mais popular e comumente consumida pela população brasileira (SM da Intervenção: 435,93 mg de sódio/g; 0,37 mg de potássio/g; 1,42 de magnésio/g; e 150 mcg de iodo/g).

### Composição dos alimentos

O consumo alimentar foi avaliado por um registro alimentar de três dias aplicado durante ambas as fases de intervenção para analisar o consumo de minerais que poderiam afetar a pressão arterial, tais como cálcio, magnésio, potássio e sódio. Os dados foram analisados utilizando-se o programa Dietbox®, baseado nas tabelas de composição de alimentos do Instituto Brasileiro de Geografia e Estatística (IBGE)^13^ e Tucunduva,^14^sendo que a segunda foi usada somente na ausência de um alimento específico nas tabelas do IBGE.^13^

### Análises de urina

Cada participante recebeu um frasco de 2 litros para coleta de urina 24 horas, e foi instruído tanto verbalmente como por escrito, para coletarem uma amostra de urina de 24 horas. A primeira urina do dia foi descartada, e todas as outras ao longo do dia foram coletadas até a primeira urina da manhã seguinte, que foi incluída, aproximadamente à mesma hora da primeira urina do dia anterior. A urina foi analisada no laboratório da Universidade Federal de Goiás usando uma técnica de membrana não seletiva.^15^

### Análise da Pressão arterial

As medidas de pressão arterial sistólica (PAS) e a pressão arterial diastólica (PAD) foram obtidas utilizando-se um aparelho digital semiautomático (OMRON 705 CPINT, Illinois, EUA), seguindo a 7ª Diretriz Brasileira de Hipertensão Arterial.^1^ Todos os pacientes realizaram monitorização residencial da pressão arterial (MRPA), seguindo a 4ª Diretriz Brasileira para MRPA.^16^ Os participantes foram instruídos a realizarem medidas de 24 horas, três pela manhã e três à tarde, por quatro dias. Os testes foram considerados válidos se o mínimo de 15 medidas corretas fosse realizado durante o período.

### Uso de sal

Os participantes receberam de um a dois quilos (dependendo da média de consumo familiar mensal) de SH ou de SM, de acordo com o grupo em que foi alocado. Após o período de *washout*, os participantes receberam a mesma quantidade do outro sal.

Os participantes foram orientados a usarem somente o sal fornecido durante a intervenção, e a devolverem o sal remanescente ao centro de pesquisa no final do período de intervenção, para se estimar o consumo médio por pessoa.

### Análise estatística

As análises estatísticas foram realizadas usando o programa SPSS para Windows, versão 20. A normalidade da distribuição dos dados foi testada usando o teste de Kolmogorov-Smirnov, que mostrou que os dados não tinham distribuição normal. Diferenças entre o momento basal e o pós-intervenção em cada grupo foram determinadas pelo teste de Wilcoxon para variáveis não paramétricas. As análises entre os grupos foram realizadas usando o teste de Mann-Whitney para variáveis não paramétricas. A ingestão de sal também foi dividida pela densidade total do nutriente, e as diferenças de ingestão entre os grupos analisadas pelo teste de Mann-Whitney. A estatística descritiva foi usada para todas as variáveis; as variáveis contínuas foram apresentadas como mediana e intervalo interquartil, e as variáveis categóricas como frequência e porcentagem.

A diferença entre os grupos foi testada pela análise por intenção de tratar e por protocolo e, uma vez que não houve diferenças entre as duas análises, somente a análise por protocolo é apresentada no estudo. Um valor de p <0,05 foi considerado estatisticamente significativo.

## Resultados

Dos 44 pacientes elegíveis, 25 concordaram em participar; sete deles não compareceram na primeira visita e, assim, 18 participantes entraram no estudo. Devido a razões pessoais, dois participantes desistiram antes do início do estudo, e 17 participantes completaram ao menos uma das intervenções. Dos 17 participantes analisados, 14 participaram dos dois braços de intervenção, um somente da intervenção com SM, e dois somente da intervenção com SH, devido a motivos pessoais (Figura 1). Nós analisamos 14 participantes, uma vez que não houve diferença entre a análise por intenção de tratar e por protocolo.

Medidas antropométricas e características demográficas estão descritas na Tabela 1.

**Tabela 1:** – Características antropométricas dos participantes do estudo (n=17)

Características basais		N=17
Idade (anos)		58 (54; 60,5)
Índice de Massa Corporal (kg/m^2^)		29,20 (27,55;35,33)
Circunferência da cintura (cm)		98 (93,50;104,75)
Média de comensais em casa		3 (2;3,37)
Tabagismo	Sim	1 (5,9%)
	Não	16 (94,1%)
Raça	Negra	4 (23,53%)
	Branca	7 (41,18%)
	Mista	6 (35,29%)
Alcoolismo	Não	17 (100%)
Nível educacional		
Ensino fundamental I		3 (17,6%)
Ensino fundamental II	Completo	2 (11,8%)
	Incompleto	1 (5,9%)
Ensino médio	Completo	5 (29,4%)
	Incompleto	2 (11,8%)
Técnico		4 (23,5%)
Renda familiar		
Nenhuma		1(5,9%)
≤ US$473		9 (52,9%)
US$473 - US$945		6 (35,3%)
> US$945		1 (5,9%)
Atividade física regular	Sim	11 (64,7%)
	Não	6 (35,3%)

*IMC: Índice de massa corporal.*

A ingestão mediana de sal por pessoa durante as intervenções com SH e SM foi 6,37g e 5,98 g, respectivamente, sem diferença significativa (p=0,808). A duração mediana da intervenção foi 35 dias em ambos os grupos.

Os valores de pressão arterial e concentrações de minerais na urina não foram significativamente diferentes após as intervenções em comparação ao momento anterior à intervenção (Tabelas 2 e 3).

**Tabela 2:** – Pressão arterial, e concentrações urinárias de sódio, potássio, e cálcio antes e após a intervenção com sal do Himalaia (n=15)

	Antes	Após	p^1^
PAS (mmHg)	118,5 (111,0,130,5)	117,5 (114,0,133,5)	0,932
PAD (mmHg)	70 (65,0; 76,0)	68,5 (66,0,79,0)	0,977
Sódio (mEq/L)	151,5 (111,00; 194,75)	159 (134,00; 192,00)	0,875
Potássio (mEq/L)	57,5 (43,50; 70,75)	55 (40,00; 74,50)	0,362
Cálcio (mEq/L)	107,5 (73,75; 175,25)	96 (57,47; 145,50)	0,423

*Valores apresentados em mediana (percentis 25 e 75). ^1^Teste de Wilcoxon para medidas não paramétricas; SH: sal do Himalaia; PAD: pressão arterial diastólica; PAS: pressão arterial sistólica.*

**Tabela 3:** – Pressão arterial, e concentrações urinárias de sódio, potássio, e cálcio antes e após a intervenção com sal de mesa (n=16)

	Antes	Após	p^1^
PAS (mmHg)	121 (111,133,00)	118 (109, 141)	0,463
PAD (mmHg)	74 (70,00; 78,00)	70 (67,00; 81,00)	0,329
Sódio (mEq/L)	158 (92,00; 191,00)	151 (116,00; 195,00)	0,345
Potássio (mEq/L)	54 (48,00; 65,00)	48 (37,00; 64,00)	0,173
Cálcio (mEq/L)	113,90 (65,70; 188,10)	84,20 (72,00; 118,50)	0,433

*Valores apresentados em mediana (percentis 25 e 75). ^1^Teste de Wilcoxon para medidas não paramétricas; SH: sal do Himalaia; PAD: pressão arterial diastólica; PAS: pressão arterial sistólica.*

**Figura 2:**
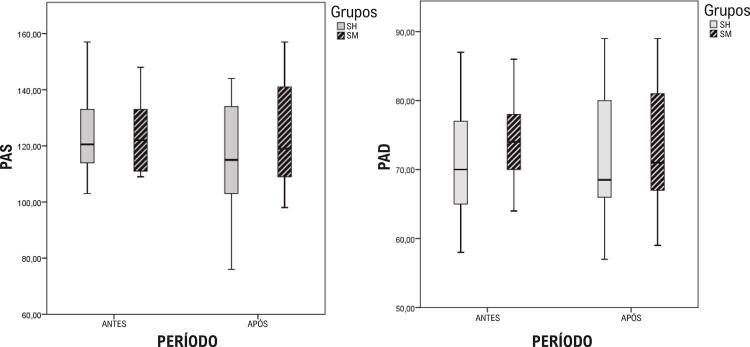
– *Comparação dos valores de pressão arterial sistólica (PAS) e pressão arterial diastólica (PAD) entre os grupos submetidos à intervenção com sal do Himalaia (SH) e sal de mesa (SM) antes e após as intervenções.1 (n=14)*

A análise dos registros alimentares não mostrou diferença significativa na ingestão de sódio, cálcio, magnésio e potássio entre as intervenções SH e SM (Tabela 4). Ainda, a análise entre grupos não evidenciou diferenças significativas na pressão arterial ou concentração de minerais entre SH ou SM antes e após a intervenção (Figuras 2 e 3).

**Tabela 4:** – Comparação da ingestão mediana de sódio, potássio, magnésio e cálcio dos participantes submetidos à intervenção com sal do Himalaia e à intervenção com sal de mesa (n=14)

	Sal do Himalaia	Sal de mesa	p^***1***^
Na (mg)	1054,07 (727,71;1607,69)	848,3 (567,52; 1390,33)	0,222
K (mg)	1652,2 (1340,41;1848,70)	1639,87 (1318,44; 2367,36)	0,485
Ca (mg)	329,11 (247,03;466,73)	363,93 (245,30; 522,66)	0,474
Mg (mg)	151,71 (125,22;178,07)	158,61 (119,00;187,52)	0,643

*Valores apresentados em mediana (percentis 25 e 75). ^1^ Teste de Mann- Whitney; Na: sódio; K: potássio; Ca: cálcio; Mg: magnésio.*

**Figura 3:**
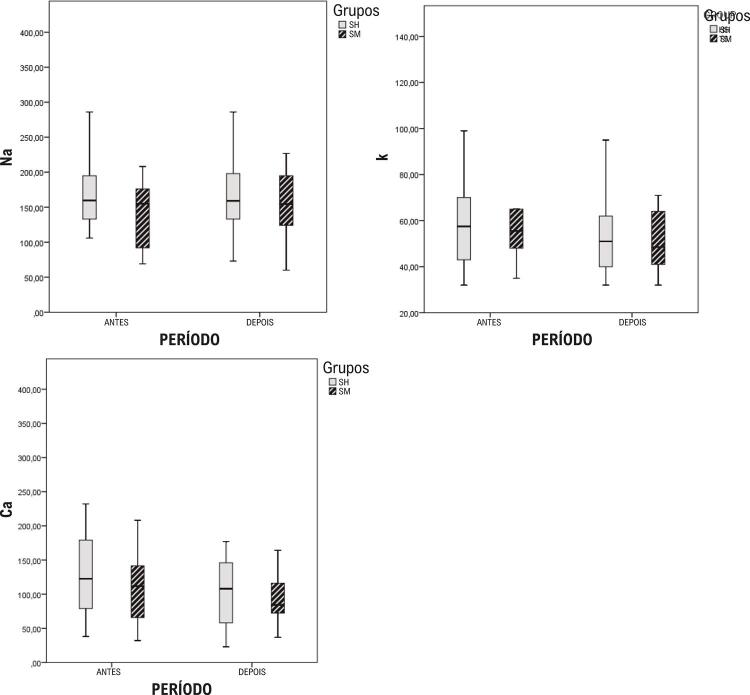
Comparação de sódio (Na), cálcio (Ca), e potássio (K) entre os grupos submetidos à intervenção com sal do Himalaia (SH) e sal de mesa (SM) antes e após as intervenções (n=14) Teste de Mann- Whitney.

## Discussão

Em nosso conhecimento, este é o primeiro estudo a investigar os efeitos da ingestão de SH sobre a pressão arterial e concentrações de minerais na urina. Os resultados sugeriram que não houve diferenças significativas intragrupo ou entre grupos antes e após as intervenções.

Em nosso estudo, após ambas as intervenções, não foram observadas alterações significativas na pressão arterial. O SH fornecido aos participantes continha 64,01 mg menos sódio por grama de sal que o SM fornecido. Considerando a média de consumo de sal em cada grupo, a média de ingestão de sódio a partir de SH foi de 2268 mg e de SM 2506 mg por dia. Portanto, a diferença média na ingestão de sódio foi 238 mg por dia, uma redução muito pequena que pode explicar a falta de significância estatística. Drake et al.^17^ também analisaram a composição do SH e o SM e não encontraram diferenças significativas na concentração de sódio (3,68 x 10^5^ versus 3,81x 10^5^ ppm, respectivamente).^17^

Barros et al.^18^ encontraram diferenças significativas na pressão arterial após a substituição do sal tradicional por sal light. No entanto, o sal light possui 260 mg menos sódio por grama de sal, resultando assim em uma redução maior na ingestão de sódio em comparação ao SH.^18^ No entanto, Arantes et al.^19^ analisaram o efeito da redução da ingestão de sódio (6g-4g) sobre a pressão arterial e concentração urinária de sódio em indivíduos hipertensos. Seus resultados estavam de acordo com os nossos; reduções na ingestão de sódio não foram associadas com mudanças significativas na pressão sanguínea.^19^

De acordo com a OMS^5^ e He et al.,^20^ há uma redução na PAS e na PAD após uma redução na ingestão de sódio da quantidade usualmente consumida pela população, 11 gramas por dia, para o valor recomendado, 5-6 gramas por dia.^5,20^ A ingestão de sódio, estimada pelo método de coleta de urina de 24 horas, foi 3,47 g após a intervenção com SH, e 3,65 após a intervenção com SM. Assim, independentemente do tipo de sal usado, o consumo foi maior que o recomendado pela OMS.^5^ Apesar de o delineamento do estudo não haver nos permitido acompanhar cada participante e garantir o uso correto de sal, a quantidade média de sal usada por pessoa não pôde explicar a concentração de sódio observada na urina. Nossa hipótese é a de que a ingestão excessiva de sódio deve-se ao consumo de alimentos ultraprocessados, os quais não foram considerados nesta análise. Arantes et al.^19^ também sugerem que a falta de controle sobre o consumo de alimentos processados e refeições feitas fora de casa provavelmente interfere nos valores de excreção urinária de sódio e pressão arterial.

Ainda, a ingestão aumentada de sódio observada pode estar relacionada a características da amostra, isto é, indivíduos hipertensos, que possivelmente preferem e consomem mais sal que indivíduos normotensos.^21^

Apesar da maior concentração de potássio no SH, o grupo que recebeu esse sal não mostrou concentrações mais altas de potássio urinário ou redução significativa na pressão arterial. Esse resultado corrobora o estudo de Barros et al.^16^ que não mostrou influência da concentração de potássio do sal light na redução da pressão arterial em indivíduos hipertensos. Uma possível razão para essa controvérsia poderia ser que a recomendação de ingestão de potássio para melhorar a pressão arterial é de 4700 mg, um valor superior que o encontrado no SH.^22^ Assim, a ingestão de potássio deveria ser encorajada por fontes alimentares, tais como verduras e frutas.

Além da ausência de diferenças significativas observada nos parâmetros clínicos entre o consumo de SH e SM, é importante notar que o SH custa até 30 vezes mais que o SM.

Este estudo apresenta algumas limitações, tais como o pequeno tamanho amostral, e a impossibilidade se de controlar a ingestão alimentar dos participantes durante o estudo. Além disso, a ingestão individual de sal pode ter sido superestimada ou subestimada pelo método usado. Ainda, a variabilidade da sensibilidade individual ao sódio não foi medida e, portanto, poderia ser uma limitação. No entanto, nossos achados destacam a necessidade de práticas com base em evidências por parte de profissionais da saúde, uma vez que nem todos os benefícios anunciados nos rótulos foram comprovados cientificamente. Mais estudos são necessários para confirmar nossos achados.

## Conclusão

Não houve diferenças significativas na pressão arterial ou na excreção urinária de sódio quando comparadas antes e após as intervenções, ou entre os grupos que receberam SH e SM. Assim, o estudo mostrou que a substituição de SH por SM não foi uma medida eficaz para melhorar os parâmetros de pressão arterial. Mudanças no estilo de vida, incluindo redução na ingestão de sal, e prática regular de atividade física, são ainda as melhores estratégias no controle da hipertensão. Existe uma clara necessidade de mais estudos controlados randomizados, incluindo uma amostra maior, para investigar o impacto do consumo do SH sobre a saúde.
