# Supplementary material for: Comparação entre os Efeitos da Ingestão de Sal do Himalaia e de Sal Comum sobre os Valores de Sódio Urinário e Pressão Arterial em Indivíduos Hipertensos
Source: Arq Bras Cardiol. 2022 Jan 11;118(5):875–82. [Article in Portuguese] doi: 10.36660/abc.20210069 (PMC9368875; doi:10.36660/abc.20210069)
Supplement: Translation

# Translation: Comparison between the Effects of Hymalaian Salt and Common Salt Intake on Urinary Sodium and Blood Pressure in Hypertensive Individuals

**Authors:** Isabela P. Loyola, Mauri Félix de Sousa, Thiago Veiga Jardim, Marcela M. Mendes, Weimar Kunz Sebba Barroso, Ana Luiza Lima Sousa, Paulo César B. Veiga Jardim

**Affiliations:** 1 Brazil Liga de Hipertensão Arterial - Universidade Federal de Goiás, Goiânia, GO – Brazil; 2 Brazil Hospital das Clínicas - Universidade Federal de Goiás, Goiânia, GO – Brazil; 3 Brazil Departamento de Nutrição - Faculdade de Ciências da Saúde de Brasília, Brasília, DF – Brazil

**Keywords:** Blood Pressure, Hypertension, Cardiovascular Diseases, Risk Factors, Sodium Chloride, Sodium Chloride,Dietary, Urinalysis

## Abstract

**Background:**

The Himalayan salt (HS) has become a popular alternative for the traditional table salt (TS) due to its health benefit claims, particularly for individuals with arterial hypertension. However, despite the increase in HS consumption, there is still a lack of clinical evidence to support a recommendation for its consumption by health professionals.

**Objective:**

This cross-over study aimed to compare the impact of HS and TS intake on systolic blood pressure (SBP) and diastolic blood pressure (DBP), and urinary sodium concentration in individuals with arterial hypertension.

**Methods:**

This study recruited 17 female patients with arterial hypertension who ate out no more than once a week. Participants were randomized into two groups, to receive and consume either HS or TS. Before and after each intervention, participants had their blood pressure measured and urine collected for mineral analysis. A p-value < 0.05 was considered statistically significant.

**Results:**

There were no statistically significant differences before and after the HS intervention for DBP (70mmHg vs. 68.5mmHg; p=0.977), SBP (118.5 mmHg vs. 117.5 mmHg; p= 0.932) and sodium urinary concentration (151 mEq/24h vs. 159 mEq/24; p=0.875). Moreover, the between-group analysis showed no significant differences after the intervention regarding SBP (117mmHg vs 119 mmHg; p=0.908), DBP (68.5 mmHg vs. 71mmHg; p= 0,645) or sodium urinary concentration (159 mEq/24h vs. 155 mEq/24h; p=0.734).

**Conclusion:**

This study suggests that there are no significant differences on the impact of HS consumption compared to TS on blood pressure and sodium urinary concentration in individuals with arterial hypertension.

## Introduction

Hypertension (HTN) is one of the main risk factors for cardiovascular disease (CVD), and affects more than 35% of the Brazilian population over 40 years old.^1^ It is well established that treating HTN may reduce the risk of cardiovascular events; therefore, this is considered to be one of the primary public health strategies for tackling CVDs.

Sodium intake is a key modifiable risk factors for HTN. ^2^ Studies show that high sodium intake is associated with higher blood pressure, while a low or moderate intake can have the opposite effect.^2^ The World Health Organization (WHO) currently recommends a sodium intake of 2 g per day;^5^ yet, in many countries salt consumption is actually more than double.^6^ In Brazil, for instance, the average sodium consumption is 4.7 grams per day, mostly from table salt (TS) and seasonings (74.4%). ^7^

Within this context, the Himalayan salt (HS) has become a popular alternative for the traditional TS, particularly for hypertensive individuals. Social media has become part of the public health scene and has been used to access, share, and spread medical information, being responsible for recent changes in health behavior.^8^ In this context of excessive media consumption, boosted by the increase of food advertisements by social media, many health benefits have been attributed to the HS, without robust scientific evidence, contributing to the HS hype.

Those who advocate for the consumption of HS to control HTN base themselves on the beneficial effects of its unrefined characteristic. The rationale is that, unlike traditional salt, HS would retain a higher concentration of minerals such as iron, magnesium, calcium, zinc, and potassium, which are inversely associated with blood pressure values.^9^

Despite the increase in HS consumption and its health claims, there is still a lack of scientific evidence to support clinical recommendations by health professionals. Therefore, this study aimed to compare the impact of HS and TS consumption on blood pressure, and calcium, sodium, and potassium urine concentrations in individuals with arterial hypertension.

## Methods

### Study design

This was a randomized crossover trial that compared the effects of HS and TS intake on urinary sodium values and blood pressure of hypertensive individuals. Women with HTN aged between 40 and 65 years old were recruited for this study from a multidisciplinary care clinic for HTN. Inclusion criteria included: residing in the metropolitan region of a Brazilian city, with no changes in antihypertensive medication for at least 60 days.

The calculations were made based on previous data on the effects of reductions in sodium intake on blood pressure.^3^ Sample size was calculated for comparison of means, considering an effect size of 1.56,^3^ an alpha of 0.05 and test power (1-β) of 90%, and the result was 10 participants in each group.

Patients with heart failure, stroke in the last six months, acute myocardium infarction in the previous three months, uncontrolled diabetes (glycated hemoglobin above 8%), liver disease, hypothyroidism, chronic kidney disease, unstable psychiatric disorders, illicit drug users, and alcoholics were excluded, as were those who had their meals prepared with a salt different from the one provided by this study more than once per week.

This study was approved by the Research Ethics Committee of General Hospital of a Brazilian University (069428/2017) and all patients signed an informed consent form. The study was conducted under the Federal Resolution 446/2012.^12^

Before and after each intervention (HS and TS), participants attended two visits, with a 3 or 4 day-interval between them, conducted by the same researcher. Before the commencement of the intervention, biochemical tests were requested for participants who did not have recent tests recorded, and anthropometric measures (weight, height, and waist circumference) and demographic characteristics of all participants included in the study were collected. At their first visit, participants were randomly assigned to use either HS or TS (Figure 1). After four weeks of intervention and an additional two-weeks of washout, participants were crossed over to the alternative salt for another four weeks of intervention. During the washout period, participants were instructed to maintain their usual diet and consume the salt they were used to.

**Figure 1:** – *Flow diagram of patients’ randomization.*

Additionally, before and after each intervention, a blood pressure device and a urine container were provided to each participant to perform blood pressure measurements and to collect a 24-hour urine sample, respectively. After three to four days, participants returned to the research center with the blood pressure device and the urine collected.

### Salt Composition

We analyzed nine HS samples and three TS obtained from food markets in a metropolitan region in Brazil, to verify iodization and minerals’ concentration. All samples of both salts were iodine fortified.

The HS brand whose sodium content was the closest to the mean of all HS samples was chosen for the intervention (intervention HS: 371.92 mg of sodium/g, 1.8 mg of potassium/g, 1.7 of magnesium/g, and 25.1 mcg of iodine/g), and the TS brand chosen was the most popular and commonly consumed by the Brazilian population (Intervention TS: 435.93 mg of sodium/g, 0.37 mg of potassium/g, 1.42 of magnesium/g and 150 mcg of iodine/g).

### Food composition

Dietary intake was evaluated using a three-day food record applied during both intervention phases, to analyze the consumption of minerals that could affect blood pressure, such as calcium, magnesium, potassium, and sodium. Data were analyzed using the Dietbox^TM^ software, based on IBGE^13^ and Tucunduva food composition tables,^14^ the latter being used only in the absence of a specific food in the IBGE tables.^13^

### Urine analysis

Each participant received a 2.0-L urine jug and was instructed, orally and in writing, to collect a 24-hour urine sample. The first urine voided in the morning was discarded, and all other urine samples throughout the day were collected until and including the first urine void of the following morning, approximately at the same time of the first urine of the previous day. Urine was analyzed at the laboratory of the Federal University of Goias using the ion-selective membrane technique.^15^

### Blood Pressure Analysis

Systolic Blood Pressure (SBP) and Diastolic Blood Pressure (DBP) measurements were obtained using a semi-automatic digital device (OMRON 705 CPINT, Illinois, USA) following the 7^th^ Brazilian Guideline of Arterial Hypertension.^1^All patients undertook Home Blood Pressure Monitoring (HBPM), following the IV Brazilian Guidelines for HBPM.^16^ Participants were instructed to perform 24 measurements, three in the morning and three in the afternoon for four days. Tests were considered valid if at least 15 effective measures were performed during the period.

### Salt dispensing

Participants received one to two kilograms (depending on monthly average family consumption) of HS or TS, according to their allocation group. After the washout period, participants received the same amount of the other salt.

Participants were instructed to use only the salt provided during the intervention and to return the remaining salt back to the research center after the intervention period, for estimation of the mean consumption per person.

### Statistical analysis

Statistical analyses were performed using the SPSS statistical program for Windows version 20. Normality of data distribution was tested using the Kolmogorov-Smirnov test, which showed that the data were not normally distributed. Differences between baseline and post intervention in each group were determined using the Wilcoxon test for the nonparametric variables. Analysis between groups was performed using the Mann-Whitney test for nonparametric variables. Sodium intake was also divided by total nutrient density and differences in intake between groups were analyzed using the Mann-Whitney test. Descriptive statistics were used for all variables; continuous variables were presented as median and interquartile range, and categorial variables as frequency and percentage.

The difference between groups was tested by intention to treat (ITT) and per protocol (PP) analysis, and since there were no differences between the two analyses, only PP analysis is shown in this study. A p-value < 0.05 was considered statistically significant.

## Results

Of 44 eligible patients, 25 agreed to participate; seven of them did not attend the first visit, thus 18 participants entered the study. Due to personal reasons, two participants withdrew before the start of the study, and 17 participants completed at least one of the two intervention arms. Of the 17 participants analyzed, 14 participants underwent both intervention arms, one only the TS intervention and 2 only HS intervention, due to personal reasons (Figure. 1). We analyzed 14 participants since there were no difference between ITT and PP analysis.

Anthropometric measurements and demographic characteristics are described in Table 1.

**Table 1:** – Anthropometric characteristics of study participants (n=17)

Baseline characteristics		N=17
Age (years)		58 (54; 60.5)
BMI (kg/m^2^)		29.20 (27.55;35.33)
Waist circumference (cm)		98 (93.50;104.75)
Average number of people eating at home		3 (2;3.37)
Smoking	Yes	1(5.9%)
	No	16 (94.1%)
Race	Black	4 (23,53%)
	White	7 (41,18%)
	Mixed race	6 (35.29%)
Alcoholism	No	17 (100%)
Education Level		
Elementary School		3 (17.6%)
Middle School	Complete	2 (11.8%)
	Incomplete	1 (5.9%)
High School	Complete	5 (29.4%)
	Incomplete	2 (11.8%)
Technical Degree		4 (23.5%)
Family income		
None		1(5.9%)
≤ US$473		9 (52.9%)
US$473 - US$945		6 (35.3%)
> US$945		1 (5.9%)
Regular physical activity	Yes	11 (64.7%)
	No	6 (35.3%)

*BMI: Body mass index.*

The median of salt intake per person during HS and TS intervention was 6.37 grams and 5.98 grams, respectively, with no significant difference (p=0.808). Median duration of the intervention was 35 days in both groups.

Blood pressure values and urine mineral concentrations after both interventions were not significantly different compared with pre-intervention (Tables 2 and 3).

**Table 2:** – Blood pressure values and sodium, potassium and calcium urine concentrations before and after the Himalayan salt intervention (n=15)

	Before	After	p^1^
SBD (mmHg)	118.5 (111.0,130.5)	117.5 (114.0,133.5)	0.932
DBP (mmHg)	70 (65.0,76.0)	68.5 (66.0,79.0)	0.977
Sodium (mEq/L)	151.5 (111.00,194.75)	159 (134.00, 192.00)	0.875
Potassium(mEq/L)	57.5 (43.50,70.75)	55 (40.00,74.50)	0.362
Calcium(mEq/L)	107.5 (73.75,175.25)	96 (57.47,145.50)	0.423

*Values are shown as median (25th,75th). 1Wilcoxon test for non-parametric measures; HS: Himalayan Salt; SBD: systolic blood pressure; DBP: diastolic blood pressure.*

**Table 3:** – Blood pressure values and sodium, potassium and calcium urine concentrations before and after the table salt intervention (n=16)

	Before	After	p^1^
SBD (mmHg)	121 (111,133.00)	118 (109,141)	0.463
DBP (mmHg)	74 (70.00, 78.00)	70 (67.00, 81.00)	0.329
Sodium(mEq/L)	158 (92.00,191.00)	151 (116.00,195.00)	0.345
Potassium(mEq/L)	54 (48.00, 65.00)	48 (37.00,64.00)	0.173
Calcium(mEq/L)	113.90 (65.70, 188.10)	84.20 (72.00, 118.50)	0.433

*Values are shown as median (25^th^,75^th^). ^1^ Wilcoxon test for non-parametric measures; HS: Himalayan salt TS: table salt; SBD: systolic blood pressure; DBP: diastolic blood pressure.*

The analysis of food records showed no significant difference in sodium (total amount p=0.222 or nutrient density, p=0.195), calcium, magnesium and potassium intake between TS and HS interventions (Table 4). Moreover, the intergroup analysis showed no significant differences in blood pressure and mineral concentration between HS and TS before and after intervention (Figures 2 and 3).

**Table 4:** – Comparison of median intake of sodium, potassium, magnesium and calcium of participants undergoing the Himalayan salt and the table salt intervention (n=14)

	Himalayan salt	Table salt	p^***1***^
Na (mg)	1054.07 (727.71,1607.69)	848.3 (567.52, 1390.33)	0.222
K (mg)	1652.2 (1340.41,1848.70)	1639.87 (1318.44, 2367.36)	0.485
Ca (mg)	329.11 (247.03,466.73)	363.93 (245.30, 522.66)	0.474
Mg (mg)	151.71 (125.22,178.07)	158.61 (119.00,187.52)	0.643

*Values are shown as median (25th,75th). 1Mann- Whitney Test; Na: sodium; K: potassium; Ca: calcium; Mg: magnesium.*

**Figure 2:** – *Comparison of systolic blood pressure (SBP) and diastolic blood pressure (DBP) values between pre-and post-interventions (Himalayan salt [HS] and the table salt [TS] interventions)^1^ (n=14)*

**Figure 3:** Comparison of urinary sodium (Na), calcium (Ca) and potassium (K) values between Himalayan salt (HS) and table salt (TS) groups before and after intervention (n=14); Mann- Whitnney Test.

## Discussion

To our knowledge, this is the first study to investigate the effects of HS consumption on human blood pressure and urine mineral concentrations. The results suggested no significant differences within or between groups before and after interventions.

In our study, after both interventions, there was no change on blood pressure values. The HS given to participants had 64.01 mg less sodium per gram of salt than the TS provided. Considering the average salt consumption in each group, mean sodium intake from HS and TS was 2268 mg and 2506 mg per day, respectively. Therefore, the average difference in sodium intake was 238 mg daily, a minor reduction that may explain the lack of significance. Drake et al.^17^ also analyzed the composition of Himalayan and table salt and did not find significant difference in sodium concentration (3.68 x 10^5^ and 3.81x 10^5^ ppm, respectively).^17^

Barros et al.^18^found significant differences in blood pressure values after the replacement of traditional salt with light salt. However, light salt has 260 mg less sodium per gram of salt, hence resulting in a greater reduction in sodium intake as compared to the HS.^18^ In contrast, Arantes et al.^19^ analyzed the effect of salt intake reduction (6g-4g) on blood pressure and urinary sodium concentration in hypertensive individuals. Their results were in line with ours; reductions of salt intake were not associated with significant changes in blood pressure.^19^

According to the WHO^5^ and He et al.,^20^ there is a decrease in SBP and DBP after a reduction in salt intake from the amount usually consumed by the population, 11 grams daily, to the recommended value, 5 to 6 grams daily.^5,20^ The estimated sodium intake using the 24-hour urine collection method sample was 3.47g after HS and 3.65g after TS intervention. Therefore, regardless of the type of salt used, consumption was higher than the recommended by OMS.^5^ Although the study design did not allow us to follow each participant to guarantee the correct use of the salt, the average amount of salt used per person could not explain the sodium concentration observed in the urine. We hypothesize that the excess sodium intake may be due to the consumption of ultra-processed foods that were not accounted in this analysis. Arantes et al.^19^ also suggest that the lack of control over the consumption of processed foods and out-of-home meals probably interferes on urinary sodium excretion and blood pressure results.^19^

Moreover, the increased sodium intake observed may be related to the characteristics of the sample, i.e., individuals with HTN, who may prefer and consume more salt than normotensive population.^21^

Despite the higher content of potassium in HS, the HS intervention group did not show higher urinary potassium concentrations or significant decrease in blood pressure. This result corroborates the study of Barros et al.,^16^ which demonstrated no influence of the light salt potassium content on blood pressure reduction amongst people with arterial hypertension. One possible reason for this controversy could be that the recommendation of potassium intake to improve blood pressure is 4700 mg, a value higher than the one found in the HS.^22^ Therefore, potassium intake should be encouraged by food sources such as vegetables and fruits.

In addition to the observed lack of significant differences in clinical parameters between TS and HS consumption, it is important to note that HS costs up to 30 times more than TS.

This study has some limitations such as the small sample size and the impossibility to control participants’ food intake during the study. Moreover, individual salt intake may have been overestimated or underestimated by the method used. In addition, the variability in individual sensitivity to sodium was not measured and therefore could be a limitation. Nevertheless, our findings highlight the need of evidence-based practices by health professionals, as not all claimed benefits on labels have been scientifically proven. Further studies are required to confirm our findings.

## Conclusion

There were no significant differences between pre- and post-interventions or between HS and TS groups in blood pressure and urinary sodium excretion. Therefore, the replacement of TS with HS was shown to be an ineffective measure to improve blood pressure parameters. Lifestyle modifications, such as reduction in salt intake along with regular exercise, remain the best strategy in arterial hypertension control. There is a clear need for more randomized controlled studies, especially with a larger sample size, to investigate the impact of HS consumption on health.
